# Theoretical Description of Changes in Conformation and Symmetry of Supramolecular Systems During the Reception of a Molecular Signal

**DOI:** 10.3390/ijms26136411

**Published:** 2025-07-03

**Authors:** Yuriy Gorovoy, Natalia Rodionova, German Stepanov, Anastasia Petrova, Nadezda Penkova, Nikita Penkov

**Affiliations:** 1 Faculty of Digital Systems, Yaroslavl State Technical University, Yaroslavl 150023, Russia; 2 OOO “NPF “MATERIA MEDICA HOLDING”, Moscow 129272, Russia; rodionovann@materiamedica.ru (N.R.); stepanovgo@materiamedica.ru (G.S.); petrovaao@materiamedica.ru (A.P.); 3 Institute of Cell Biophysics, Federal Research Center “Pushchino Scientific Center for Biological Research of the Russian Academy of Sciences”, Pushchino 142290, Russia; kokanchik@rambler.ru (N.P.); nvpenkov@rambler.ru (N.P.)

**Keywords:** supramolecular chirality, chiral self-assembly, distant molecular signal, highly diluted solution, signal transfer, supramolecular systems, molecular recognition, emission spectrum, symmetry

## Abstract

Aqueous solutions are not homogeneous and could be considered supramolecular systems. They can emit electromagnetic waves. Electromagnetic emission from one supramolecular system (“source”) can be received by another supramolecular system (“receiver”) without direct contact (distantly). This process represents a transfer of a “molecular signal” and causes changes in conformation and symmetry of the “receiver”. The aim of the current work is to theoretically describe such changes primarily using a solution of the chiral protein interferon-gamma (IFNγ) as an example. We provide theoretical evidence that supramolecular systems of highly diluted (HD) aqueous solutions formed by self-assembly after mechanical activation generate a stronger molecular signal compared to non-activated solutions, due to their higher energy-saturated state. Additionally, molecular signals cause supramolecular systems with complex (including chiral) structures to undergo easier changes in conformation and symmetry compared to simpler systems, enhancing their biological activity. Using statistical physics, we obtained the parameter *Ic*, characterizing the magnitude of conformational and symmetry changes in supramolecular (including chiral) systems caused by molecular signals. In quantum information science, there is an analogue of the parameter *Ic*, which characterizes the entanglement depth of quantum systems. This study contributes to the understanding of the physico-chemical basis of distant molecular interactions and opens up new possibilities for controlling the properties of complex biological and chemical systems.

## 1. Introduction

Modern research in the field of supramolecular chirality and self-assembly of molecular systems occupies a central position at the intersection of physical chemistry, biophysics, molecular biology, and nanotechnology. These areas allow us to deepen our understanding of the principles of organization of matter and the mechanisms of interaction of substances [1,2,3,4,5,6,7,8,9,10]. In addition, these studies are important for the fields of biomedicine and pharmaceuticals. For example, in the works [11,12], the production of new biomaterials with enhanced biological functions and biocompatibility is discussed. Chirality manifests itself everywhere, including at the molecular level in the organism: receptors and enzymes are chiral formations and interact with chiral molecules, including drugs [13].

During the interaction of a receptor or enzyme with a ligand (let’s call it a “direct” interaction), a change in the conformation of molecules occurs [14,15,16,17]. Molecule’s hydration shells play an important role in such interaction [18,19,20,21]. However, recently, several studies have shown the existence of so-called “distant” interaction between aqueous solutions of substances that are supramolecular systems [22,23,24,25,26,27,28,29]. As a result of the “distant” interaction, the so-called “molecular signal” from the “source” is transmitted to the “receiver” [27,28] (hereinafter we will call it the “distant molecular signal,” DMS), changing the conformational, structural and dynamic properties of the “receiver” [23], and, consequently, its symmetry. It is assumed that the “source” generates an electromagnetic field with specific parameters, which is involved in the process of transmitting the molecular signal to the “receiver” [23,30,31,32]. Interestingly, water by itself can also act as a “receiver” [24,33]. In the current article, we perform a theoretical analysis of the reception of DMS by the “receiver”.

By now, a number of facts characterizing the reception of DMS by supramolecular systems have been established experimentally. For example, some works demonstrate the dependence of the reception of DMS by supramolecular systems of aqueous solutions on the external magnetic field [24,25,29]. In the current work, the effect of an external magnetic field on aqueous solutions is not considered separately, but the special role of the geomagnetic field will be described below.

It should be noted that several studies on the reception of DMS from highly diluted (HD) solutions obtained using mechanical activation (shaking) have been published. This technology increases the energy saturation and nonequilibrium state of metastable HD solutions, which is accompanied by supramolecular self-assembly. As a result, the intensity of their intrinsic emission increases [25,34] and, as a consequence, the effect of the DMS of these solutions also increases [24,25,26,27,28,29].

The concept of a supramolecular matrix describes the possibility of forming symmetrical supramolecular systems in HD aqueous solutions due to the technological processing of these solutions [35,36]. The change in the structure, conformation, and symmetry of supramolecular systems during their interaction can be interpreted as a process of reception of a “molecular signal”. In the present work, the ideas presented in [35,36] are significantly further developed.

By now, the physico-chemical properties of HD aqueous solutions have been studied quite well. These solutions may not contain molecules of the initial substance, but they contain supramolecular systems, the nuclei of which may be nanosized bubbles (bubstons) [37] and/or various impurities [38,39]. The special physico-chemical properties of HD aqueous solutions are linked to nanoassociates, which are supramolecular components of aqueous solutions. Nanoassociates practically disappear when the solution containing them is shielded from the geomagnetic field [40]. In addition, a frequency of 7.85 Hz has been identified at which nanoassociates are formed in HD solutions under conditions of geomagnetic field shielding [41]. When interacting supramolecular systems are shielded from the geomagnetic field, DMS transfer disappears or at least significantly weakens [24,25,29].

HD solutions have an important property: during technological processing, they acquire some physico-chemical properties of supramolecular systems of the initial substances and can then transfer these properties to biological systems [35,36,42,43]. The theoretical possibility of implementing such an effect is substantiated by mechanochemical methods [44]. We note that distant interactions are not related to heat transfer, since many experiments were carried out under isothermal conditions [22,23,24,25,26]. Solutions and suspensions containing systems with a more complex structure have their intrinsic emission with a more complex spectrum. These systems are more susceptible to DMS [26,35,45]. With an increase in the duration of distant exposure to supramolecular systems of aqueous solutions, the magnitude of the effect increases [23,24,27,28,29].

Thus, the significant role of the geomagnetic field in the reception of DMS is already well known. The presence of the geomagnetic field maintains a metastable energy-saturated state of supramolecular systems of aqueous solutions and thus provides DMS with energy from supramolecular systems of aqueous solutions.

HD aqueous solutions have been used as a source of DMS. It has been shown that shaking, which is carried out during the preparation of HD aqueous solutions, significantly changes the physico-chemical (including magnetic) properties of these solutions [46]. Mechanochemical activation creates a metastable energy-saturated state in the HD solution, which is a prerequisite for supramolecular self-assembly.

During DMS reception, the influence of heat exchange is excluded, and the “source” performs work on the “receiver” without direct contact due to its intrinsic electromagnetic emission. Intrinsic emission from solutions containing supramolecular systems has been demonstrated both experimentally and in calculations. For example, Montagnier et al. [47,48] demonstrated that HD DNA solutions emit electromagnetic radiation in the ULF range (500–3000 Hz) [47,48]; Penkov and Penkova detected emission in the IR range (400–7500 cm^−1^) from protein solutions, HD protein solutions, and HD buffer solutions [22,49]; and Elia et al. [33] discussed emission from HD fullerene solution. Moreover, even from water, it is possible to record intrinsic emission under certain conditions in the visible [50], IR [22,25], or THz range [51]. Theoretical calculations also confirm the possibility of emission from supramolecular systems [52].

The question of DMS reception by biological systems has not been clarified to date. However, it is well known that living systems at the molecular, cellular, tissue, and organism levels are susceptible to magnetic [53,54,55] and electromagnetic fields of different frequency ranges. For example, THz radiation is known to affect various biological objects: individual cells, animal and plant cells, proteins, and nucleic acids [56,57,58,59]. In addition, it has been demonstrated that near-THz radiation (the wavelength is 81.5 µm, the radiation frequency is 3.6 THz) causes conformational changes in albumin, and as a consequence, changes its functional state, namely, it reduces the binding capacity of albumin with a native ligand [60]. Pulsed microwave radiation changes the protein conformation, reducing the percentage of alpha-helical and beta-pleated structures, and the changes correlate with the radiation dose [61]. Radiofrequency radiation (2.4 GHz) stimulates differentiation of pluripotent stem cells into cardiomyocytes, neurons, and skeletal muscle cells [62], and nonthermal radiofrequency stimulation modulates neuronal activity and affects the function of the nervous system at the level of neural circuits [63]. Even weak impacts can influence biological systems [53,64]. Therefore, emissions from supramolecular systems of one biological system can distantly (i.e., through DMS transfer) affect another supramolecular system.

There has been a growing number of DMS transfer examples. In 1923, Gurvich discovered the ultraweak glow of biological objects, called mitogenetic radiation [65]. It was demonstrated that the ultraviolet component of mitogenetic radiation stimulates cell division [66,67,68]. Foletti et al. (2017) [31] claimed that it is possible to transmit an electromagnetic signal from one biological system to another and described a process that puts this idea into practice. Thomas et al. (2000) [69] presented experimental data on the transfer of drug properties to biological systems through an electromagnetic field. Rossi et al. (2011) [70] described electromagnetic signal transfer between cells. Montagnier et al. (2011) [47] developed a model based on quantum field theory, with the help of which they describe the induction of low-frequency electromagnetic waves in HD aqueous solutions by some DNA sequences of viruses and bacteria.

Recent experimental studies have established the similarity of the results of the direct effect of supramolecular systems of HD solution of interferon-gamma (IFNγ) on chiral IFNγ [71] and the transfer of DMS from HD solution of IFNγ to IFNγ [26].

The aim of the current work is to theoretically describe changes in conformation and symmetry upon DMS reception, primarily using a solution of the chiral protein interferon-gamma (IFNγ) as an example. We believe that the results obtained in our work will clarify the peculiarities of the process of DMS reception by supramolecular systems.

## 2. Results

### 2.1. Description of the Reception of Direct Action of Supramolecular Systems and Their DMS in the Reaction of Molecular Recognition: Increase in Biological Activity

Direct interaction between molecules (“guest” and “host”) is manifested in their selective binding without the formation of covalent bonds. Such interaction, called molecular recognition, is studied by supramolecular chemistry [72]. To implement molecular recognition, it is necessary to have specific binding sites in which the “guest” and “host” molecules must be complementary, i.e., correspond to each other both structurally and energetically.

As noted above, the analogy of the result of the direct action of supramolecular systems on a solution of molecules (i.e., the “guest” on the “host”) and the result of the action of DMS from a supramolecular system on a solution of molecules (i.e., the “source” on the “receiver”) has been shown [26,71]. This analogy is clearly demonstrated in the examples of molecular recognition reactions in which the supramolecular systems of HD aqueous solutions (or supramolecular systems of solutions treated with DMS from HD solutions) act as “guests” and biological systems act as “hosts”. Biological systems are also supramolecular systems, since biological macromolecules function mainly in an aqueous environment [24]. This analogy can be revealed by parameters characterizing the reaction of molecular recognition of supramolecular systems [72]. Such parameters are the selective capacity and free energy of complexation [73].

**Figure 1:**
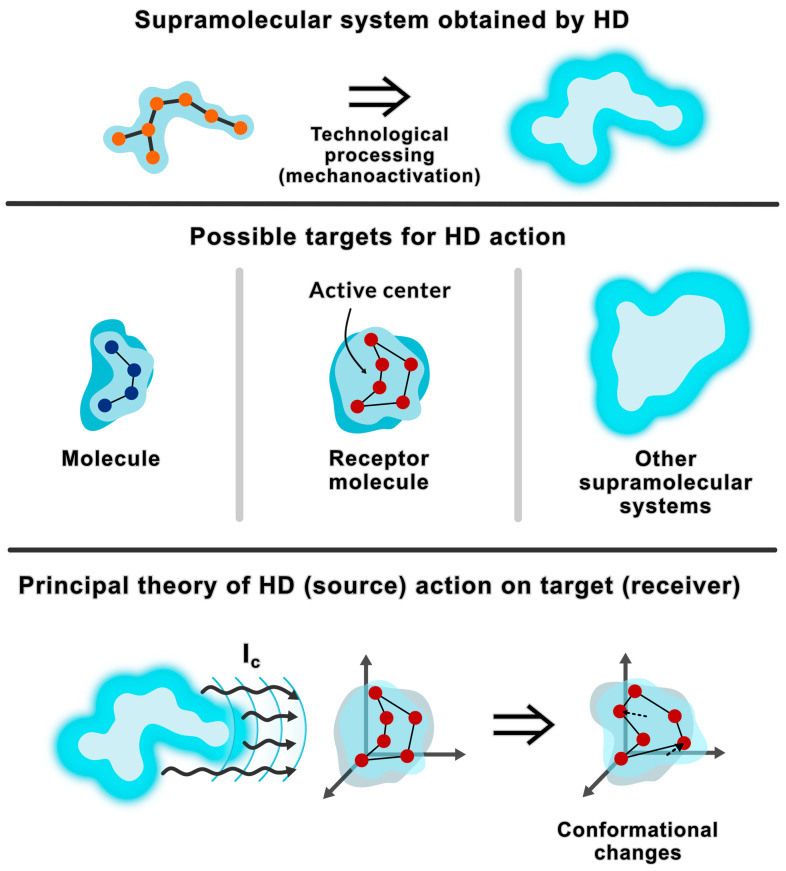
Schematic representation of a supramolecular system in HD aqueous solutions and the result of its DMS action on the “receiver”. Upper panel: supramolecular systems are formed in the process of self-assembly in HD aqueous solutions obtained by technological processing (including mechanical activation). Middle panel: molecules, molecular receptors, and supramolecular systems are potential targets for the action of DMS. Bottom panel: change in the conformation of the target (using the example of a molecular receptor) due to its reception of DMS from supramolecular systems: a change in the conformation of the active center in the protein molecule is shown, which leads to a change in the conformation of the entire protein molecule.

Figure 1 shows a model according to which, in HD aqueous solutions, after mechanical activation and in the process of self-assembly, supramolecular systems are formed that affect the “receiver” through DMS transfer.

In a molecular recognition reaction with direct interaction of supramolecular systems, the selective capacity is defined as the ratio of the binding constants for the studied and control ligands. The lower limit of the selective capacity can be determined by the ratio of the yields of the molecular recognition reaction [73].

*s* ˃ *C_I_*/*C_C_*, (1)

where *s* is the selective capacity of the molecular recognition reaction, *C_I_* is the yield of the reaction with the studied ligand, and *C_C_* is the yield of the reaction with the control ligand.

The free energy of the complex formation can be calculated (more specifically, its upper limit can be determined) by knowing the selective capacity as follows:

∆G^0^ ˂ −*RTln*(*s*), (2)

where ∆G^0^ is the Gibbs free energy of complex formation, *R* is the gas constant, *T* is the complex formation temperature, and *s* is the entropy.

The work [74] describes the results of an experimental study of the molecular recognition reaction using a model of mature human adipocyte culture, which is directly (with contact) exposed to supramolecular systems of the HD solution. The yield of the reaction was determined by the level of phosphorylation of the beta subunit of the insulin receptor. Two “guests” were used in the work: (1) insulin (control) and (2) an antihyperglycemic drug, Subetta, with subsequent addition of insulin. Note that the active substance of Subetta is an HD solution of antibodies to the beta subunit of the insulin receptor (along with an HD solution of antibodies to endothelial NO synthase). This preparation was obtained as a result of mechanical activation, underwent supramolecular self-assembly, and has a chiral supramolecular system. It turned out that the yield of the reaction in the case of using Subetta increased by 1.8 times compared to the control. Therefore, the selective capacity (*s*_1_) of this reaction is ˃1.8, and the free energy of complex formation (∆G^0^_1_) is ˂−1.4 kJ/mol [73]. An increase in the reaction yield means an increase in the biological activity of insulin as a result of the preceding action on the receptor of a contact influence from Subetta.

The paper [24] presents the results of an experimental study of DMS transfer based on the yield of the molecular recognition reaction. In this case, the molecular recognition reaction was studied using a mouse neutrophil model, and the yield of the reaction was assessed by the production of reactive oxygen species (ROS). Two different experimental setups were used in this study. In one of them, the “guest” in the molecular recognition reaction was a supramolecular system in IFNγ solution that previously received DMS from HD solution of antibodies to IFNγ for different periods of time. Moreover, since the HD solution of antibodies to IFNγ was obtained using mechanical activation accompanied by supramolecular self-assembly, it is most likely that it has a chiral supramolecular system, as does the IFNγ molecule itself. As a control, a solution of IFNγ that had not previously received DMS was used. It was shown that with a DMS exposure duration of 20 min, the reaction yield increased by 1.8 times compared to the control. Therefore, the selective capacity (*s*_3_) is ˃1.8, and the free energy of complex formation (∆G^0^_3_) is ˂−1.4 kJ/mol. With a DMS exposure duration of 60 min, the reaction yield increased by 2.0 times compared to the control [24]. Therefore, in this experimental setup, the selective capacity (*s*_4_) is ˃2.0, and the free energy of complex formation (∆G^0^_4_) is ˂−1.6 kJ/mol. An increase in the reaction yield means an increase in the biological activity of the supramolecular systems participating in the molecular recognition reaction (i.e., IFNγ solution) as a result of preliminary exposure to DMS (i.e., the intrinsic electromagnetic emission of HD solution of antibodies to IFNγ).

In a different experimental setup, supramolecular systems of water participated in the molecular recognition reaction as a “guest” after transferring DMS from the HD solution of antibodies to IFNγ for various periods of time. Water that did not receive DMS was used as a control. It was found that with a DMS exposure duration of 20 min, no difference was detected with the control, whereas with a DMS exposure duration of 60 min, the reaction yield increased by 1.8 times compared to the control. Consequently, the selective capacity (*s*_2_) in this reaction is ˃1.8, and the free energy of complex formation (∆G^0^_2_) is ˂−1.4 kJ/mol. An increase in the reaction yield means an increase in the biological activity of the supramolecular systems participating in the molecular recognition reaction (i.e., water) as a result of preliminary exposure to DMS (i.e., the intrinsic electromagnetic emission of HD solution of antibodies to IFNγ).

It can be noted that the use of solutions having a chiral supramolecular system for the transfer of DMS to the “guest”, which then participates in a molecular recognition reaction, increases the effectiveness of such a reaction.

IFNγ solution turned out to be a more sensitive “receiver” of DMS from HD solution of antibodies to IFNγ compared to another “receiver”—water. This is logical, since with spatial correspondence (complementarity) of the structures of the “source” and “receiver”, the susceptibility of the “receiver” to the effects of DMS can be enhanced as a result of the resonance of their intermolecular vibrations. For direct interaction, this was proven earlier [73].

Another important note: experiments have shown that with an increase in the duration of exposure to DMS, its effect is enhanced [23,24,27,28,29], i.e., the “receiver” more effectively recognizes the “source” during long-term distant interaction.

The results presented in this section allow us to conclude that the increase in the yield of the molecular recognition reaction and, consequently, the increase in biological activity are associated with the reception by the “host” of molecular signals from the supramolecular systems of HD solutions. This occurs both during their direct (contact) interaction and during the transfer of DMS. It is important that the source of molecular signal in the experiments presented is chiral supramolecular systems obtained as a result of mechanical activation followed by supramolecular self-assembly.

### 2.2. Parameter Ic Characterizes the Change in Structure, Conformation, and Symmetry (Including Chirality) of Supramolecular Systems

The theoretical description of direct (contact) action on the “host” and transfer of DMS to the “receiver” is similar. Theoretical analysis of these processes is based on a single method: the method of statistical physics of complex systems. Theoretical analysis of contact interaction of complex (supramolecular) systems is presented in [73]. In this work, Liouville’s theorem and an analogue of Noether’s theorem are proven for a complex system in a metastable state. A complex system consists of several interacting subsystems and, as a result of this interaction, has a deformed conformation and symmetry.

Let us formulate the physical meaning of the description of the deformation of the structure and symmetry of a supramolecular system during the transition of this system from one conformation to another using the method of statistical physics of complex systems. Statistical physics uses not a three-dimensional space to describe a system consisting of many particles, but a multidimensional Gibbs phase space [75], the dimension of which is 6N, where N is the number of particles in the system. In this case, each particle in the Gibbs phase space has six coordinates: three spatial coordinates and three momentum projections. The change in the state of the system is described by the trajectory of one point in the multidimensional Gibbs phase space. Instead of analyzing this trajectory, subject to ergodicity conditions, the Gibbs phase ensemble is analyzed: a set of identical systems located at different points of the trajectory at the same moment in time. The phase ensemble occupies a certain volume of the Gibbs phase space.

The characteristic of the state of the system is not the trajectory of the system in the Gibbs phase space, but the volume of this phase space occupied by the phase ensemble. The restrictions imposed on the motion of the particles of the system, i.e., the presence of the structure of the system, are manifested in the topology of the phase volume of the system. Thus, for an ideal gas consisting of non-interacting particles, the phase volume is absolutely symmetrical. The interaction of particles means the deformation of the phase volume. The interaction of subsystems of a complex system consisting of many particles means a multidimensional deformation of the phase volume.

Changes in the structure, conformation, and symmetry of a supramolecular system can be expressed through changes in the entropy and phase volume of this system, since in the equilibrium and metastable states, the entropy of the system is proportional to the logarithm of the phase volume.

In [73], it was shown that there is a parameter, *Ic*, that characterizes the depth of deformation of the structure and the change in conformation and symmetry of a complex (supramolecular) system. This parameter can be expressed through the deformation of the Gibbs phase volume or the difference between the entropy of a complex system and the total entropy of non-interacting subsystems of this system.

*Ic* = *S_c_* − (*S*_1_ + *S*_2_) = *lnG_c_* − (*lnG*_1_ + *lnG*_2_), (3)

where *S_c_* is entropy of a complex system, *S*_1_ and *S*_2_ are entropies of the first and second subsystems (in a non-interacting state) that make up this complex system, *G_c_* is Gibbs phase volume of a complex system, *G*_1_ and *G*_2_ are Gibbs phase volumes of the first and second subsystems (in a non-interacting state) that make up this complex system.

Thus, the parameter *Ic* has a thermodynamic and statistical–physical meaning. According to Liouville’s theorem for a complex system [73], in a metastable state of this system, the parameter *Ic* remains constant, i.e., the value of the parameter *Ic* changes when the system transitions from one metastable state to another (transition from one conformation of the complex system to another). According to Noether’s theorem for a complex system [73], the parameter *Ic* is invariant for canonical transformations of coordinates and momenta of a complex system. A special case of canonical transformations is a change in coordinates and momenta during the motion of the system itself. A change in the parameter *Ic* means a change in the nature of the system’s motion (deformation of its Gibbs phase space), i.e., a change in the symmetry of the complex system [73]. Since in the statistical physics of complex systems there are no restrictions on the type of interaction of subsystems, the conclusions of this theory are applicable to both contact and distant interactions of subsystems. Particular cases of such interaction are the reception of direct (contact) influence of the “guest” on the “host”, as well as the transfer of DMS from the “source” to the “receiver”. Each of the participants in these processes is a supramolecular system.

Both direct (contact) and distant influence of one system on another leads to a change in the structure, conformation, and symmetry of the complex (supramolecular) system to which it is directed. Here, an analogy with heat exchange is appropriate, which can occur in different forms: contact (conductive and convective) and distant (radiative heat exchange). Therefore, in order to characterize the direct (contact) influence or reception of DMS by supramolecular systems, it is necessary to calculate the change in the parameter *Ic*. The following sections provide examples of such calculations for DMS.

A special role in biology is played by chiral biological molecules, which, together with water molecules, form supramolecular systems. In this regard, it is important to emphasize that the parameter *Ic* characterizes the change in the structure, conformation, and symmetry of chiral supramolecular systems. There is direct experimental confirmation of this. Andronikashvili et al. [76] measured the heat capacity of DNA molecules with their aqueous environment (salmon DNA) in the native and unwound state. The measurements were conducted in the temperature range from 4.5 K to 300 K, which made it possible to calculate the entropies of chiral supramolecular systems: native and unwound DNA with their hydration shells. The relationship between entropy (*S*) and heat capacity (*c*(*T*)) is expressed by the following formula:

$$S=\int_{0}^{T}\frac{c(T)}{T}dT,$$

where *T* is temperature.

The change in chirality during unwinding of DNA molecules can be quantitatively expressed as the difference in entropies (3) of native and unwound molecules. The entropy of unwound molecules is 2.9% higher than the entropy of DNA molecules that have retained their symmetry. This means that the value of the parameter *Ic* constitutes 2.9% of the entropy of unwound DNA molecules. This work experimentally shows that at 0 °C, the hydration shell of native DNA remains in a liquid state and does not crystallize. Consequently, the thesis that the parameter *Ic* may characterize the change in structure, symmetry, and conformation (including chirality) of supramolecular systems has not only a theoretical basis, but also experimental confirmation.

Thus, in [76], the entropies of two supramolecular systems are compared: native DNA molecules with their hydration shell and unwound DNA molecules with the number of water molecules equivalent to the hydration shell of the native molecules. The entropy of native molecules is less than the entropy of unwound molecules, since native molecules have a more complex structure and a more deformed (in this case, reduced) Gibbs phase volume, which corresponds to Formula (3).

### 2.3. Analysis of the Reception of a Distant Molecular Signal by Supramolecular Systems Using Spectral Methods

#### 2.3.1. Analysis of the Reception of a Distant Molecular Signal by the Parameters of the Dielectric Spectrum in the Terahertz Region

This section presents an analysis of experimental data obtained in the study of IFNγ solution with a chiral structure and HD solution of antibodies to IFNγ, during the preparation of which supramolecular self-assembly took place. We calculated the parameter *Ic* during the reception by the “receiver” (IFNγ solution) of DMS from the “source” (lactose solution saturated with HD solution of antibodies to IFNγ) [19]. The dielectric spectrum of IFNγ solution in the terahertz (THz) region was measured at different durations of signal exposure. As a control, the spectra of the “receiver” were measured, which was exposed to another “source”—lactose saturated with technologically treated water (i.e., lactose not containing HD solution of AT to IFNγ).

In the THz range, the parameters of the dielectric spectrum are determined by the formula for approximating the spectrum of dielectric permittivity. In the described experiment, we were particularly interested in the following parameters: the amplitude *A* of the resonant oscillations and the resonant frequency of the intermolecular bonds *ϖ*. Knowing these parameters, we can estimate the value of *Ic.*

The article [73] describes a model of a supramolecular system as a system of coupled oscillators. When calculating the Gibbs phase volume (*G*) of such a system, it is necessary to consider that its value for a one-dimensional oscillator is proportional (i.e., equal to within a constant factor) to the product of the oscillation frequency and the square of the amplitude of these oscillations (4) [77]:

*G* ≈ *ωA*^2^, (4)

The value of *Ic* is equal to the difference of the natural logarithms of the Gibbs phase volumes (2,3), i.e., it is determined with an accuracy of up to a constant. Consequently, the value of *Ic* can be expressed as (5).

*Ic* = *ln*(*ωA*^2^) − *ln*(*ω_c_A*^2^*_c_*), (5)

where *ω* is the resonant frequency of the oscillations of the intermolecular bonds of the receiver, *A* is the amplitude of the resonant oscillations of the intermolecular bonds of the receiver, *ω_c_* is the resonant frequency of oscillations of intermolecular bonds in the control, and *A_c_* is the amplitude of resonant oscillations of intermolecular bonds in the control.

The calculation results (in arbitrary units) are provided for the durations of distant exposure of 3 and 4 h:

3 h: *Ic* = 0.68 ± 0.12

4 h: *Ic* = 0.78 ± 0.21

For shorter durations of distant exposure, the spectrum parameters are indistinguishable from the control (*Ic* = 0).

The results of the experiment, both in the author’s interpretation [23] and in the interpretation presented in this article, showed a noticeable change in the spectral characteristics of IFNγ solution as a result of its reception of DMS, as well as a noticeable (although not very distinct) dependence of these spectral characteristics on the duration of DMS. Calculations of the change in the value of *Ic* indicate a change in the structure, conformation, and symmetry of the aqueous solution of IFNγ as a result of its interaction with DMS, and these changes possibly occur in intermolecular bonds of supramolecular systems.

Let us note the correspondence of the results of this experiment to our concept. The effects of DMS from two supramolecular systems on one “receiver”, also a supramolecular system, are compared. DMS from a source with a structure complementary to the “receiver” has a more significant effect on the “receiver”. The effect manifested itself in the amplification of the resonant oscillations of the “receiver” due to the work performed by DMS (there is no heat exchange between the “source’ and the “receiver”). The work is performed due to the nonequilibrium state of the “source”, to which it is brought by mechanochemical technological processing. The change in the amplitude of the resonant oscillations is manifested in the increase in the Gibbs phase volume.

#### 2.3.2. Analysis of the Reception of a Distant Molecular Signal Using the Spectra of Electromagnetic Emission in the IR Region

In this section, similarly to the previous one, an analysis of the experimental data obtained during the study of the chiral solution of IFNγ and the HD solution of antibodies to IFNγ is presented. *Ic* calculations are also presented here, based on data from measurements of the intensity of the intrinsic electromagnetic emission of aqueous solutions of IFNγ after their reception of DMS [22]. The following substances were used as “sources” of DMS: water prepared using HD technology, including mechanical activation (technological control); HD buffer solution (glycine); HD IFNγ solution; and HD solution of antibodies to IFNγ. The role of the “receiver” of DMS (receiver of electromagnetic emission from the “sources”) was played by IFNγ solution, whose spectrum of intrinsic IR emission (in the range of 500–2000 cm^−1^) was recorded upon exposure to various “sources”. This spectrum reflects the conformational features of the supramolecular system. The measurements were carried out using a specialized, highly sensitive experimental setup [49].

It is necessary to calculate the value of the parameter *Ic* as the difference between the entropy of a complex system and the total entropy of the subsystems that make up this complex system (3). A change in the entropy of a system is possible either as a result of heat exchange or as a result of work (when the system transitions to a metastable state). Since the conditions in the experiment, the results of which we rely on [22], were isothermal, heat exchange between the source and receiver can be neglected. Consequently, work must be achieved to transition the “receiver” from one metastable state to another (i.e., from one conformation to another). In the article [24], it is shown that the geomagnetic field supports metastable states and, as a consequence, the exchange of DMS between supramolecular systems is possible.

Heat exchange with the thermostat (environment) does not affect the metastable state of the system (except for large temperature fluctuations). Otherwise, the existence of a metastable state would be impossible. Therefore, heat exchange of the system with the thermostat can be neglected when calculating the parameter *Ic*, and the microcanonical distribution can be used to calculate the change in entropy. The following calculation formula is based on the classical definition of entropy change in statistical physics [78]:

$$∆S=ln\frac{∆G}{{\left(2\hbar \right)}^{d}},$$

where ∆S is the change in the entropy of the system, ∆G is the change in the phase volume of the system as a result of the work performed during the exchange of signals, ℏ is Planck’s constant, and *d* is the number of degrees of freedom of the system.

A system of coupled oscillators with heterogeneous oscillations (including librational and bending) may serve as a model of a supramolecular system [73]. The change in the structure of the system of coupled oscillators and, as a consequence, the change in the spectrum of its intrinsic emission can be described as a deformation of the Gibbs phase space. In turn, the deformation of the Gibbs phase space can be described as a change in the parameter *Ic*.

The logarithm of the phase volume is equal to the entropy of the system in a metastable state. Therefore, the above formula is valid for a semiclassical system. The intrinsic emission was measured in the IR range (from 500 cm^−1^ to 2000 cm^−1^), where the energy levels are sufficiently dense (the photon energy *hγ* is much less than the thermal motion energy *kT*) for the semiclassical approximation to be applicable. Thus, in calculations according to the model we propose, it is possible to use the data from Penkov’s article [22], namely the values of the difference in emission intensities of aqueous HD solutions compared to the technological control (water treated using HD technology, including mechanical activation).

To within a constant factor, the change in the intensity of monochromatic emission is as follows:

(6) $$∆I_{i}=∆n_{i}\hbar k^{2},$$

where ∆$I_{i}$ is the change in the intensity of monochromatic emission compared to the control, ∆$n_{i}$ is the difference in the number of emitted photons of a certain frequency compared to the control, ℏ is Planck’s constant, *k* is the wave vector, and *i* is the number of the component of the emission spectrum of HD aqueous solution. Consequently, *Ic* for each HD aqueous solution, accurate to a constant factor, will be equal to the following:

(7) $$Ic=\sum_{i}\frac{I_{i}}{k^{2}}$$

Since the intensity of the intrinsic emission was measured in arbitrary units [22], it makes sense to analyze only relative changes in the parameter *Ic*. As a denominator of this relative change, it is logical to choose the *Ic* of the intrinsic emission of the technological control.

The first experiment [22] was related to the IR spectra of the intrinsic emission of the “sources” of distant action, which are HD solutions. Let us designate the parameters *Ic* of the intrinsic emission of the solutions as follows: the solution prepared without the initial substance is *Ic_tech.con_*, HD buffer (glycine) is *Ic_buf_*, HD IFNγ solution is *Ic_IFN_*, and HD solution of antibodies to IFNγ is *Ic_Ab to IFN_*.

The relative values of the parameter *Ic* of the intrinsic emission of the DMS “sources” subjected to mechanical activation (irrespective of the spectrum of the intrinsic emission of the DMS “receiver” (IFNγ solution)) are as follows:

*∆Ic_buf_*/*Ic_tech.con_* = −0.077;

∆Ic_IFN_/Ic_tech.con_ = −0.047;

*∆Ic_Ab to IFN_*/*Ic_tech.con_* = −0.010

where *∆Ic_buf_* = *Ic_buf_* − *Ic_tech.con_*; *∆Ic_IFN_* = *Ic_IFN_* − *Ic_tech.con_*; *∆Ic_Ab to IFN_* = *Ic_Ab to IFN_* − *Ic_tech.con_*.

According to the experimental data presented in the article [22], the difference in the spectral intensities of the intrinsic emission ∆I was presented as a value free of errors. Two standard deviations were subtracted from the average difference in spectral intensities (over six measurements), which made it possible not to calculate the error in calculating *Ic*, since this error had already been taken into account. The experimental results show that the technological control is in the most energy-saturated nonequilibrium state with the highest intensity of intrinsic emission. Therefore, technological control, as a source of DMS, has the largest Gibbs phase volume. The other solutions are inferior to it. Except for the technological control, the most energy-saturated is the HD solution of antibodies to IFNγ, and the least is the HD solution of buffer (glycine buffer).

Note that the results of estimating the *Ic* parameter of lactose granules, obtained by differential scanning calorimetry, also showed that the technological control is the most energy-saturated compared to other HD solutions [73,79]. Thus, in [22] it has been shown that the technological processing of the initial solutions, carried out during the preparation of HD solutions, brings HD solutions into energy-saturated metastable states, and these states differ depending on the initial substance. Since the intrinsic electromagnetic emission of the “source” solutions differs, the DMS that these “sources” transfer to the “receiver” also differs.

In the second series of experiments [22], the spectra of the intrinsic emission of the “receiver” (IFNγ solution) were obtained, which was previously exposed to DMS from the above-described HD “source” solutions.

As a denominator of the relative change in the parameter *Ic* for the second series of experiments, it is logical to choose the parameter of the intrinsic emission *Ic* of the IFNγ solution exposed to the signal from the technological control–*Ic_tech_._con_*. Let *Ic* denote the intrinsic emission of IFNγ solution exposed to the signal of other solutions as follows: technological control—*Ic_tech.con_*_._, HD of the buffer (glycine)—*Ic_buf_*, HD IFNγ solution—*Ic_IFN_*, HD solution of antibodies to IFNγ—*Ic_Ab to IFN_*.

The following are the relative values of parameters *Ic*:

*∆Ic_buf_*/*Ic_tech.con_* = −0.014;

*∆Ic_IFN_*/*Ic_tech.con_* = −0.012;

*∆Ic_Ab to IFN_*/*Ic_tech.con_* = 0.019

where *∆Ic_buf_* = *Ic_buf_* − *Ic_tech.con_*; *∆Ic_IFN_* = *Ic_IFN_* − *Ic_tech.con_*; *∆Ic_Ab to IFN_* = *Ic_Ab to IFN_* − *Ic_tech.con_*.

A seemingly paradoxical result was obtained: the most energy-saturated and most complementary to the “receiver” “source” (HD solution of antibodies to IFNγ) does not increase, but decreases the “receiver’s” intrinsic emission compared to the effect of a technological control that is albeit energy-saturated but not complementary to the “receiver”. In this case, a violation of collective oscillations of hydrogen bonds (librational oscillations) is assumed, which is reflected in the IR spectrum of the “receiver’s” intrinsic emission [22]. There is an explanation for this paradox: the effect of the signal deforms the spectrum of the “receiver’s” intrinsic emission [25]. By decreasing the intensity in one range of the spectrum, it increases the intensity in another range due to the formation of new collective degrees of freedom. A decrease in the intensity in the IR region of the “receiver’s” spectrum is compensated for by an increase in the intensity of its emission in the THz region (see Section 2.3.1).

Calculations of the change in the relative value of *Ic* indicate a change in the structure, conformation, and symmetry of the aqueous solution of IFNγ (“receiver”) as a result of the influence of DMS on it.

The correspondence of the results presented in Section 2.3.1 to our concept can be interpreted as follows. The Gibbs phase volume of the “receiver” is deformed as a result of the DMS effect. This deformation can be multidirectional: the amplitudes of oscillations of one frequency can increase, while those of another frequency can decrease. Long-wave collective oscillations (in the THz range) of the “receiver” particles increase the amplitude of oscillations, while short-wave more individual oscillations (in the IR range) decrease the amplitude.

### 2.4. Measure of the Depth of Entanglement in Quantum Systems

The existence of an analogue of the Gibbs phase volume deformation depth in quantum information science reinforces the results presented in the previous sections, thanks to a unified method for describing classical and quantum systems in statistical physics. The characteristic of the behavior of a quantum system (an analogue of the Gibbs phase volume) is the density matrix (*ρ*) [78,80]. The quantum analogue of both classical entropy and Shannon entropy is the von Neumann entropy (*S*(*ρ*)).

*S*(*ρ*) = −*Sp*((*ρ*)*ln*(*ρ*)), (8)

where *Sp*((*ρ*)*ln*(*ρ*)) is the trace of the matrix ((*ρ*)*ln*(*ρ*)).

The von Neumann entropy of a complex quantum system consisting of two subsystems, a and b, is as follows:

*S*(*ρ_ab_*) = −*Sp*((*ρ_ab_*)*ln*(*ρ_ab_*)), (9)

The interaction of quantum subsystems leads to entanglement, that is, to the interdependence of the states of these subsystems.

For quantum systems, the following parameters serve as a measure of the entanglement of a complex quantum system [81]:

*Ic* = *S*(*ρ_ab_*) − (*S*(*ρ_a_*) + *S*(*ρ_b_*)), (10)

The result of entanglement of the state of a complex quantum system is similar to the result of interaction of subsystems of a classical complex system, i.e., deformation of the density matrix (change in structure, conformation, and symmetry of the complex system). Thus, the parameter *Ic* for classical and quantum systems has a single physical meaning. Note that in the experiment presented in Section 2.2 and in quantum information science, the interaction of subsystems leads to a decrease in the entropy of a complex system.

In the article [73], Liouville’s theorem and an analogue of Noether’s theorem for a classical (non-quantum) complex system were proven. The result of Liouville’s theorem for a classical complex system is as follows: the parameter *Ic* in a metastable state, i.e., with an unchanged configuration (structure, conformation, and symmetry) of a complex (supramolecular) system, remains constant. The parameter *Ic* changes when a complex system transitions from one state to another, i.e., when the configuration of a complex system changes. With regard to contact influence or reception of DMS from one supramolecular system by another supramolecular system, the parameter *Ic* characterizes the depth of changes of the supramolecular system of the “host” or “receiver” under the influence of the “guest” or “source”.

Noether’s theorem for a classical complex system is that the symmetry of a complex supramolecular system is preserved if the system is in a certain metastable state, i.e., with an unchanged configuration of the complex system. When the conformation of a complex (supramolecular) system changes, the symmetry of this system is broken. The general result of these theorems is that *Ic* is a parameter that determines the change in structure, conformation, and symmetry of a classical complex system.

#### 2.4.1. Liouville’s Theorem for a Complex Quantum System

To assess the depth of entanglement of the states of subsystems that make up a complex quantum system, the von Neumann entropies of non-interacting subsystems are compared with the von Neumann entropy of an entangled system (complex quantum system) [80,82].

Since both the complex quantum system itself (after interaction) and the subsystems that comprise it (before interaction) are in a metastable (quasi-stationary) state, the von Neumann entropy does not depend on time [80]. If:

*S*(*ρ_ab_*) = *const*,

i.e., the von Neumann entropy of a complex system ab does not depend on time;

*S*(*ρ_a_*) = *const*,

i.e., the von Neumann entropy of subsystem a does not depend on time;

*S*(*ρ_b_*) = *const*,

i.e., the von Neumann entropy of subsystem b does not depend on time;

then and
  *I_c_* = *const*.

Changes in *Ic* occur as a result of changes in the interaction energy of the subsystems that make up a complex quantum system and, as a consequence, lead to changes in the structure of this system; in this case, the complex quantum system passes from one metastable state to another.

#### 2.4.2. Analogue of Noether’s Theorem for an Entangled Quantum System

It was proven [73] that *Ic* is invariant with respect to canonical transformations that do not change the Hamilton equations describing the states of the classical system. A special case of canonical transformations is the change in coordinates and momenta during the system’s motion itself [77], i.e., during the system’s motion along its trajectory in the Gibbs phase space. Deformation of the Gibbs phase space disrupts the system’s trajectory and changes the value of *Ic*; therefore, a change in the value of *Ic* characterizes a change in the symmetry of the complex system.

The analogue of canonical transformations for quantum systems is unitary transformations [83]. The von Neumann entropy is invariant with respect to unitary transformations [81]. If:

*S*(*ρ_ab_*) = *inv*,

*S*(*ρ_a_*) = *inv*,

*S*(*ρ_b_*) = *inv*,

then and

*Ic* = *inv*.

A change in *Ic* means a deformation of the density matrix (*ρ*), that is, a change in the symmetry of a quantum complex system.

The fact that *Ic*, which we obtained and used to describe contact influence or reception of DMS from one supramolecular system by another supramolecular system, is used in quantum information science to estimate the depth of entanglement of complex quantum systems [81], supports our theoretical concepts. According to these concepts (in accordance with experimental data and the Liouville and Noether theorems proven for classical and quantum complex systems), direct (contact) influence or transfer of DMS to supramolecular systems leads to a change in their conformation and symmetry. The depth of this change can be determined using the parameter *Ic*.

## 3. Discussion

The main result presented in this article is a theoretical description of the change in structure, conformation and symmetry of supramolecular systems during contact influence or reception by one supramolecular system (“host” or “receiver”, respectively) of DMS from another supramolecular system (“guest” or “source”, respectively). The theoretical analysis does not address the issue of the interaction of supramolecular systems of aqueous solutions with an external magnetic field that maintains the metastable state of these systems. A possible mechanism for such interaction appears to be similar to the mechanism of a photochemical reaction, the efficiency of which depends on the intensity of the acting electromagnetic field, absorption, polarization, and exposure time:

*n* = *n*_0_ *e*^−^*^σIt^*

where *n*_0_ and *n* are the amount of substance at the initial moment of time *t*_0_ and after time *t*, respectively, *σ* is the absorption cross-section, *I* is the emission intensity, and *t* is the exposure time.

However, in the process of DMS transfer, the acting electromagnetic field (generated by the “source”) has rather low energies (IR and THz radiation). Although one might expect that such low energies would not have any significant effect on the properties of the “receiver”, for example, the conformation of proteins, a number of works have already been published showing that both the conformation of proteins [57,58,59,60,61] and the macroproperties of living objects change under the influence of IR and THz radiation [56,62,63]. It is important to note that the radiation intensities could even be noticeably less than 1 mW/cm^2^ [56].

The change in protein conformation under the influence of low energies is explained by the fact that they are in the equilibrium of the population of states of the protein conformational landscape [84]. Even low energy exposure can lead to a shift in this equilibrium. Local changes in protein structure can change the functional activity of the protein [84]. In particular, a shift in equilibrium can be caused by a change in the parameters of protein hydration [85].

The theoretical description of distant interaction is based on the methods of the statistical physics of complex systems [73]. We obtained the invariant *Ic*, which characterizes the depth of deformation of the Gibbs phase space of a complex system as a result of the interaction of its subsystems. This parameter describes the deformation of the structure and the change in the symmetry of a complex (supramolecular and chiral) system as a result of either contact interaction with another supramolecular system or reception of DMS from it. Evidence for the validity of this description was obtained by analyzing previously published [23,86] experimental data on the IR and THz spectra of supramolecular systems (solutions of IFNγ, which has a chiral structure), participating in the reception of DMS (Section 2.3.1 and Section 2.3.2).

The proposed method has some limitations. The parameter *Ic* quantitatively characterizes the depth of change in the conformation and symmetry of supramolecular systems only during their transition from one metastable state to another metastable state. The results of experiments and calculations are given for small changes in the conformations of supramolecular systems (i.e., weak changes in the energy spectrum).

## 4. Materials and Methods

The article presents the results of a theoretical analysis of previously published experiments. References to these articles with experimental data and detailed descriptions of the materials used in the experiments are presented in Section 2 [18,19,20,51].

The following methods of theoretical analysis are used in the article: chemical thermodynamics of supramolecular systems [49,50], statistical physics of complex (supramolecular) systems [50,52,57], and quantum information science [57,58,59]. Since the methods of theoretical analysis described in this article are adapted to the specific properties of supramolecular systems and are themselves results, they are presented in Section 2.

## 5. Conclusions

The results of experiments and calculations based on these results, presented in Section 2, indicate the possibility of using the parameter *Ic* as a measure of change in the chirality of a supramolecular system. Using chemical thermodynamic methods, quantitative correspondence of the parameters of the reaction of molecular recognition of supramolecular systems under contact and distant influence of other supramolecular systems has been proven (Section 2). Previously, other authors have shown experimentally, and in this work we have theoretically substantiated, that supramolecular systems with a more complex (including chiral) structure more easily change their conformation, symmetry and increase their biological activity upon reception of direct (contact) or distant influences from other supramolecular systems compared to systems with a relatively simple structure.

The parameter *Ic* has an analogue in quantum information science, which characterizes the depth of entanglement (i.e., interaction) of quantum systems. The theorems of Liouville and Noether, proven for quantum systems, confirm that the quantum parameter *Ic* characterizes the change in symmetry of an entangled quantum system.

DMS transfer is a new mechanism of intermolecular communication, which brings a new level of complexity to the analysis of biological processes. The phenomenon of DMS transfer must be considered when analyzing molecular recognition reactions. This phenomenon can find application in medicine, biotechnology, and pharmacology, since DMS can enhance the biological activity of “guest” substances. Other applications may include diagnostics of pathogens that can be developed based on the detection of the influence of their electromagnetic signal on a sensor (“receiver”). Additionally, it is possible to use DMS to control the self-assembly of functional molecular structures. In general, the study of the DMS transfer process connects such fundamental sciences as physics, chemistry, and biology into an interdisciplinary paradigm, opening up new opportunities for managing complex systems from molecular interactions to cellular regulation.

## Data Availability

Data is contained within the article.

## Abbreviations

The following abbreviations are used in this manuscript:

DMS	Distant molecular signal
HD	Highly diluted
IFNγ	Interferon-gamma
